# The First Evidence of the Beneficial Effects of Se-Supplementation on In Vitro Cultivated Olive Tree Explants

**DOI:** 10.3390/plants10081630

**Published:** 2021-08-09

**Authors:** Luca Regni, Maurizio Micheli, Alberto Marco Del Pino, Carlo Alberto Palmerini, Roberto D’Amato, Simona Lucia Facchin, Franco Famiani, Alessandro Peruzzi, Hanene Mairech, Primo Proietti

**Affiliations:** Department of Agricultural, Food and Environmental Sciences, University of Perugia, Via Borgo XX Giugno 74, 06121 Perugia, Italy; alberto.delpino@unipg.it (A.M.D.P.); carlo.palmerini@unipg.it (C.A.P.); roberto.damato@unipg.it (R.D.); simonafacchin88@gmail.com (S.L.F.); franco.famiani@unipg.it (F.F.); alessandro.peruzzi93@gmail.com (A.P.); mairech.hanene@gmail.com (H.M.); primo.proietti@unipg.it (P.P.)

**Keywords:** selenium supplementation, in vitro culture, *Olea europaea*, micropropagation

## Abstract

Selenium is an essential micronutrient that provides important benefits to plants and humans. At proper concentrations, selenium increases plant growth, pollen vitality, the shelf life of fresh products, and seems to improve stress resistance; these effects can certainly be attributed to its direct and indirect antioxidant capacity. For these reasons, in the present work, the effects of selenium at different dosages on in vitro cultivated olive explants were investigated to observe possible positive effects (in terms of growth and vigor) on the proliferation phase. The work was carried out on four different olive cultivars: “San Felice”, “Canino”, “Frantoio”, and “Moraiolo”. The explants were cultured in aseptic conditions on olive medium (OM), with the addition of 4 mg·L^−1^ of zeatin, 30 g·L^−1^ of sucrose, and 7 g·L^−1^ of agar. The experimental scheme included a comparison between explants grown with five different concentrations of Na_2_SeO_4_ (0, 10, 20, 40, and 80 mg L^−1^) added to the medium during three successive subcultures. Interesting information has emerged from the results and all varieties responded to different concentrations of Selenium. The optimal Se dosages varied for each cultivar, but in general, Se concentration between 10 and 40 mg L^−1^ increased fresh and dry weight of the explants and shoot lengths. Se treatment induced in all cultivars and for all dosages used an increase in total Se content in proliferated explants. Furthermore, as the subcultures proceeded, the ability of the explants to absorb Se did not diminish. The Se content ranged from 8.55 to 114.21 µg kg^−1^ plant DW in ‘Frantoio’, from 9.83 to 94.85 µg kg^−1^ plant DW in ‘Moraiolo’, from 19.84 to 114.21 µg kg^−1^ plant DW in ‘Canino’, and from 20.97 to 95.54 µg kg^−1^ plant DW in ‘San Felice’. In general, the effect of selenium tends to decrease with the progress of subcultures and this suggests a sort of “adaptation” effect of the explants to its presence. The present study highlights for the first time the possibility of using in vitro cultures as biotechnological support to study supplementation with selenium and its effects on in vitro olive plant growth.

## 1. Introduction

Selenium (Se) is a microelement that is a component of some important enzymes involved in very specific biological roles including glutathione peroxidases, iodothyronine deiodinases, and thioredoxin reductases [1]. Its effects depend on the concentration, the chemical form, and other environmental factors [2]. In particular, regarding the concentration, it should be noted that Se at trace/moderate concentrations is important for its antioxidant activity in humans and may play a role in antioxidative mechanisms in plants [3] while elevated concentrations can be toxic [4,5]. The toxicity of Se at elevated concentrations (more than 1 mg kg^−1^) in plants can be attributed to its pro-oxidative effects as well as to metabolic disturbance [6,7]. Se, because of its chemical similarity to sulfur (S), is generally taken up by the plant via sulfate transporters and then metabolized via the S assimilation pathway [8], which may incorporate this micronutrient into organic forms such as selenocysteine (SeCys) and selenomethionine (SeMet). Recently, interest in seleno-amino acids has increased because they integrate the active centers of several selenoenzymes, which are involved in the biological synthesis and plant metabolism, and are used for protein structure determinations [9]. The interaction between selenium uptake and that of some nutrients such as nitrogen and phosphorus has been extensively studied, but the results in the literature are not unambiguous [10]. In any case, it seems that adequate administrations of nitrogen and phosphorus promote selenium uptake in plants while at high doses selenium uptake is inhibited [10].

Some of the positive effects of Se on tree plants are: promotion of plant growth, alleviation of UV-induced oxidative stress, increased antioxidative capacity of senescing plants, and regulation of the water status of plants exposed to drought and reduction of the deleterious effects of the heat stress [11,12,13]. Particularly, in olive trees (*Olea europaea* L.), several beneficial effects of Se administered as sodium selenate via foliar spray at a concentration of 100–150 mg L^−1^ in pots and field experiments and of 10–30 mg L^−1^ added to the nutrient solution in hydroponics have been reported. Some of the reported effects are: improved drought and salt stress tolerance [14,15,16], increased oil phenol content [17,18], better pollen germination [19,20], and increased Se content in fruits [21,22]. However, no studies have been done on the possible beneficial effects of Se on olive tree in vitro. Micropropagation is used worldwide because it has many advantages with respect to conventional propagation systems such as cutting and grafting (i.e., high genetic and sanitary quality of the propagated material, the possibility to produce a huge number of plants in a small space and in a short period) [23]. For these reasons, nowadays, micropropagation is used for the multiplication of rootstocks and cultivars in several fruit species [24,25]. Micropropagation has also been improved for olive tree propagation to obtain high-quality plantlets, especially some varieties that are interesting from a commercial point of view, but have a low rooting potential [26,27]. Indeed, micropropagation allows overcoming some of the inconveniences (i.e., inefficiency with certain species and/or cultivars, link with the seasonal trend, high cost, and need of wide spaces) related to the conventional asexual propagation methods; mainly cutting and grafting are used for olive trees. Moreover, in vitro cultures have always been an excellent tool to support the various laboratory techniques used to conduct genetic, microbiological, physiological, and biochemical studies. The possibility of having plant material with juvenile characters, able to develop in a short time and, therefore, to provide fast responses to various treatments is very interesting. In this context, the present work aimed to assess, for the first time to our knowledge, the effects of different doses of Se, administered in the form of sodium selenate (Na_2_SeO_4_) on the in vitro proliferation of olive explants.

## 2. Results

‘San Felice’

Considering the average values of the full set of three subcultures in the ‘San Felice’ cultivar, it emerges that Se treatment increased the total dry weight (DW) of explants (Table 1). In particular, at the dosages of 20 mg L^−1^ and 40 mg L^−1^, total DW values of 823.54 mg and 830.66 mg were observed, which were higher than those of the proliferated explants not treated (control).

**Figure 1:**
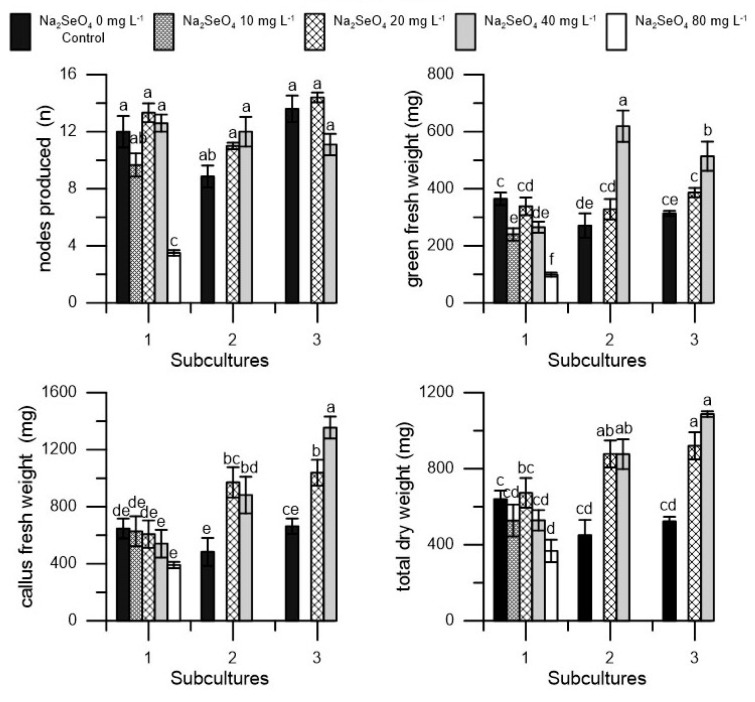
Nodes (n), green fresh weight (mg), callus fresh weight (mg), and total dry weight (mg) in ‘San Felice’ cv. in three different subcultures (1, 2, and 3) at five dosages of Na_2_SeO_4_ (0, 10, 20, 40, and 80 mg L^−1^) during the first subculture and at three dosages of Na_2_SeO_4_ (0, 20, and 40 mg L^−1^) during the second and third subculture. Each value represents the average of three replicates ± SEM. Mean values followed by different letters were significantly different (*p* < 0.05).

Considering the values monitored at the end of each subculture, in the ‘San Felice’ cultivar, the dosage of 80 mg L^−1^ already from the first subculture reduced the number of nodes, green fresh weight, and total dry weight of proliferated explants (Figure 1). In the second and third subcultures, on the other hand, an increase in green fresh weight, callus fresh weight, and consequently total dry weight was observed as a result of treatment with Se (20 and 40 mg L^−1^) (Figure 1).

‘Moraiolo’

Considering the mean values of the full set of three subcultures, the proliferated shoots of ‘Moraiolo’ showed no significant differences attributable to the Se treatment for all parameters considered (Table 2).

**Figure 2:**
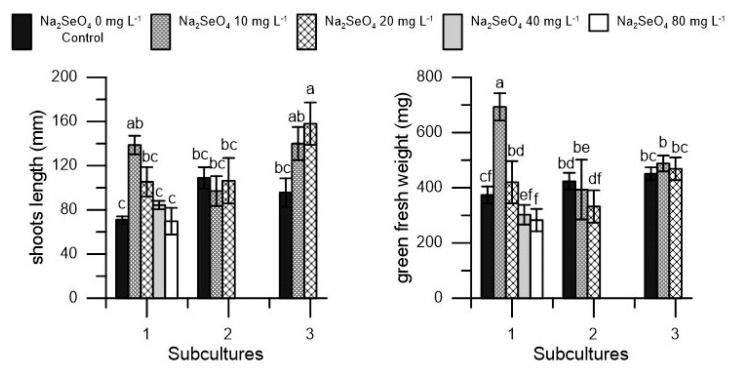
Shoot length (mm) and green fresh weight (mg) in ‘Moraiolo’ cv. in three different subcultures (1, 2, and 3) at five dosage of Na_2_SeO_4_ (0, 10, 20, 40 and 80 mg L^−1^) during the first subculture and at three dosages of Na_2_SeO_4_ (0, 10, and 20 mg L^−1^) during the second and third subculture. Each value represents the average of three replicates ± SEM. Mean values followed by different letters were significantly different (*p* < 0.05).

At the end of the first subculture, treatment with Se increased shoot length at a dosage of 10 mg L^−1^ and green fresh weight at dosages of 10 and 20 mg L^−1^. The 40 mg L^−1^ and 80 mg L^−1^ dosages resulted in reductions in green fresh weight and for this reason, they were not used in subsequent subcultures (Figure 2). In the second and third subcultures, no differences attributable to Se treatment emerged except for a slight increase in shoot length in the third subculture (Figure 2).

‘Frantoio’

The results of the averages of the three subcultures for the parameters examined on proliferated explants of the ‘Frantoio’ cultivar showed no differences attributable to treatment with Se (Table 3). The two highest Se concentrations were eliminated, denoting a probable greater susceptibility of this variety to the treatment.

**Figure 3:**
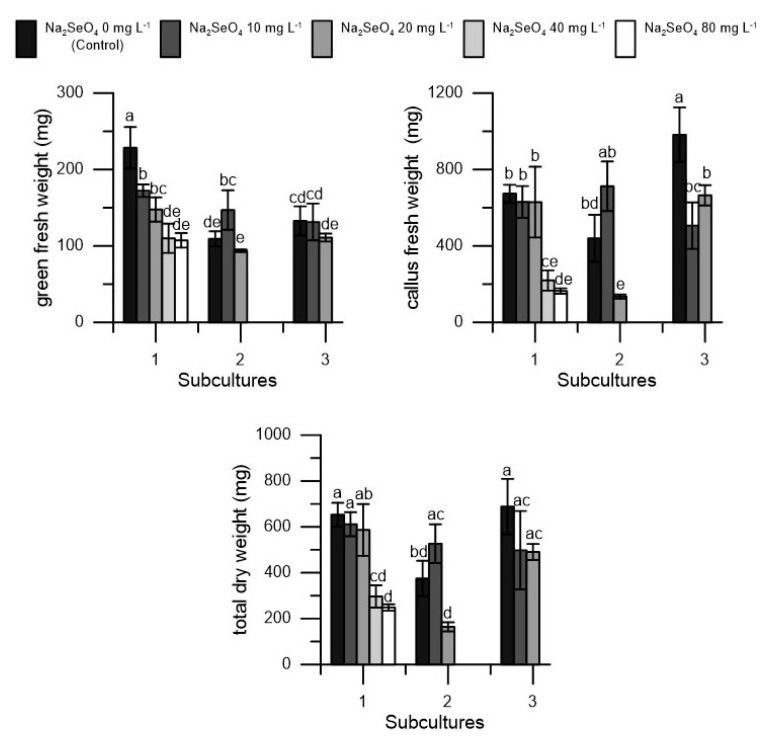
Green fresh weight (mg), callus fresh weight, and total dry weight (mg) in ‘Frantoio’ cv. in three different subcultures (1, 2, and 3) at five dosages of Na_2_SeO_4_ (0, 10, 20, 40 and 80 mg L^−1^) during the first subculture and at three dosages of Na_2_SeO_4_ (0, 10 and 20 mg L^−1^) during the second and third subculture. Each value represents the average of three replicates ± SEM. Mean values followed by different letters were significantly different (*p* < 0.05).

In the first subculture, no significant positive effects of Se treatment were observed on ‘Frantoio’ shoots. On the contrary, as stated above, the higher dosages (40 mg L^−1^ and 80 mg L^−1^) produced negative effects on explant growth and, therefore, were discarded in the subsequent subcultures (Figure 3). A slight decrease in green fresh weight was also observed in the first subculture for the 10 and 20 mg L^−1^ dosages. In the second subculture, a slight increase in green fresh weight and total dry weight was observed at 10 mg L^−1^ dosage and a decrease in callus fresh weight, especially at the 20 mg L^−1^ in the second and third subcultures (Figure 3).

‘Canino’

Considering the mean values of the full set of three subcultures for the cultivar ‘Canino’, an increase in the green fresh weight of the explants proliferated at the dosage of 40 mg L^−1^ (496.89 mg) was observed that was significantly higher than the values observed in the control (355.00 mg) and the explants treated with 20 mg L^−1^ (316.56 mg) (Table 4).

**Figure 4:**
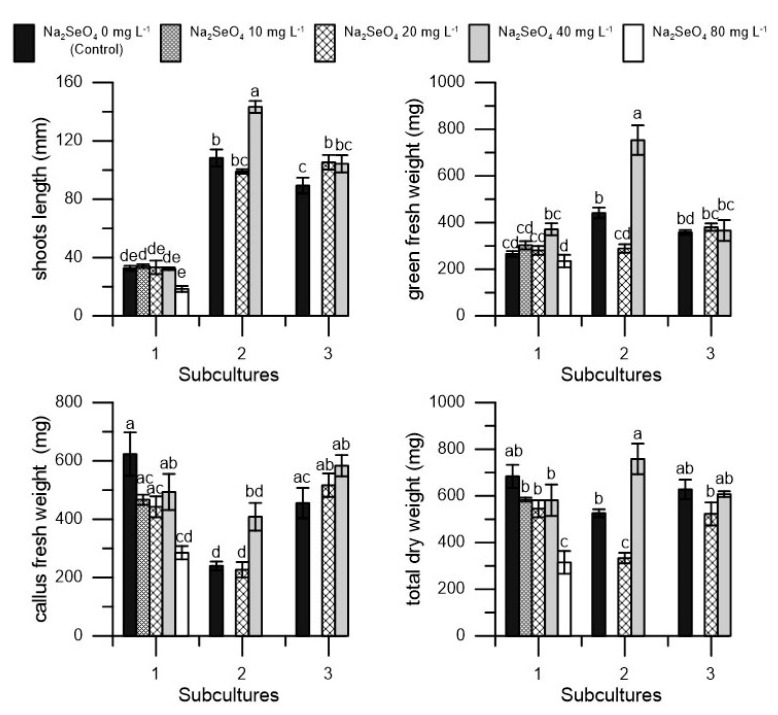
Shoot length (mm), green fresh weight (mg), callus fresh weight, and total dry weight (mg) in ‘Canino’ cv. in three different subcultures (1, 2, and 3) at five dosages of Na_2_SeO_4_ (0, 10, 20, 40, and 80 mg L^−1^) during the first subculture and at three dosages of Na_2_SeO_4_ (0, 20, and 40 mg L^−1^) during the second and third subculture. Each value represents the average of three replicates ± SEM. Mean values followed by different letters are significantly different (*p* < 0.05).

In the second subculture, treatment with Se at a dosage of 40 mg L^−1^ induced an increase in shoot length, green fresh weight, and dry weight in the proliferated explants (Figure 4). In the third subculture, however, no differences emerged for the parameters detected between the Se-treated and control explants (Figure 4).

Total Se content

In general, the treatment-induced in all cultivars and for all dosages used an increase in total Se content in proliferated explants (Table 5). Total Se contents greater than about 94 µg kg^−1^ plant DW produced deleterious effects on explant proliferation for all cultivars. As subcultures proceeded, the ability of explants to absorb Se did not decrease (Table 5).

## 3. Discussion

In the present study, a sodium selenate dosage of 80 mg L^−1^ reduced the growth of explants for all four olive cultivars examined. Some studies have reported that a Se dosage of around 1.0 mg kg^−1^ of soil is toxic to plants and causes severe yield reductions [28]. This was probably due to a phytotoxic effect exerted by too high a concentration of Se as confirmed by the total Se content of the proliferated explants determined at the end of each subculture. Excessive Se concentration is toxic for plants since the misincorporation of SeCys and SeMet into proteins impairs their function [29,30,31,32] and also because it causes oxidative and nitrosative stresses and, therefore causes metabolism disorders and damage to the cellular structures [29,30,31,32]. On the other hand, although Se is not an essential element for plants, there is evidence that in some circumstances, it can be beneficial for their growth and survival [33]. This biphasic response (better plant growth at low Se concentrations and growth inhibition and toxic effects at high concentration) is called hormesis and is common for different toxic elements [34].

Studies conducted on olive trees in the open field have shown a beneficial effect of Se in terms of increased tolerance to water stress when distributed by foliar spray at concentrations equal to or greater than 100 mg L^−1^ [14,15], while in hydroponics, concentrations up to 30 mg L^−1^ conferred an increased tolerance to salt stress [16]. In our study, the cultivar ‘San Felice’ showed significant fresh and dry weight increases with treatments of 20 and 40 mg L^−1^ of sodium selenate. However, the effects of sodium selenate at the abovementioned dosages manifested differently among the three subcultures. In the cultivar ‘Canino’, on the other hand, in the first and second subcultures with 40 mg L^−1^ of sodium selenate, a higher fresh weight of the explants was recorded, but by the third subculture, the differences were no longer appreciable. In the cultivar ‘Moraiolo’, Se favored, in the first subculture, a greater fresh and dry weight of the explants and a greater length of the shoots, but at lower concentrations (10 mg L^−1^) than in ‘San Felice’ and ‘Canino’. In this case, the differences attributable to the treatment with sodium selenate were attenuated and then disappeared in the second and third subcultures. On the other hand, the administration of sodium selenate had negative effects in the ‘Frantoio’ cultivar at the higher dosages of 40 and 80 mg L^−1^. In the literature, the processes involved in Se accumulation, and especially the synthesis of organic compounds containing Se, have been correlated with improved growth and increased antioxidant capacity [34,35,36]). In particular, proper Se forms and concentrations delivered during plant growth result in increased amounts of free amino acids, proteins, and higher defenses against oxidative stress [10,36,37]. The data obtained therefore suggests that there is a different response for each cultivar to sodium selenate treatment, demonstrating again how the effect of genotype on the performance of olive shoots reared in vitro is relevant [38,39]. Differences among cultivars and during different subcultures do not seem to be attributable to the different Se content absorbed by explants as demonstrated by the data of total Se content of proliferated explants. As expected, the Se dosage that can be used for olive trees in vitro is lower than the dosage used in open fields [14,15,17,18]. The cultivars that in the field showed a greater tolerance to unfavorable conditions, namely ‘San Felice’, ‘Canino’, and ‘Moraiolo’, seemed to respond better to treatments with sodium selenate. The obtained results show that in some olive cultivars, an appropriate amount of Se (administered as sodium selenate) can significantly improve the proliferation phase of the in vitro cultivation. In particular, the Se treatment improved the vigor of the proliferated olive shoots that had a higher fresh and dry weight compared to the untreated shoots.

Improvements in the explants’ growth of several olive cultivars have been obtained in terms of multiplication rate and quality of developed shoots also with the addition of other substances such as BAP or dikegulac to growth media containing zeatin [40,41] or coconut milk in combination with BAP without zeatin [42]. Our results show that sodium selenate, which is the most bioavailable form of Se [33], exerts in certain doses and for certain cultivars, a positive effect on the growth of in vitro proliferated explants. In addition, obtaining more robust explants is very important for the subsequent rooting phase and transplanting under ex vitro conditions since the more vigorous the plants, the better they are in developing effective roots and adapting to adverse environmental conditions [43].

## 4. Materials and Methods

### 4.1. Plant Material and Growing Conditions

The experiment was conducted on two cultivars of national importance (“Frantoio” and “Moraiolo”) and two of local importance for Central Italy (“San Felice” and “Canino”). All initial explants were represented by 10–15 mm long binodal portions excised from proliferated shoots grown on olive medium (OM) [44]. To maintain the greatest possible uniformity of the starting material, the apical portions were not used, since it is known that the apical bud is more vigorous and, even in vitro, could give rise to dominance phenomena.

Glass vessels (500 mL capacity) were used in the experiment, each containing seven explants with two leaves and 100 mL of the basal OM enriched with sucrose (30 g L^−1^), agar (7 g L^−1^) and zeatin (4 mg L^−1^), buffering the pH to a value of 5.5. The medium and vessels were autoclaved at 115 °C for 20 min, before being used in aseptic conditions under a horizontal laminar flow cabinet. The cultures were placed for 45 days in a growth chamber, characterized by a constant temperature of 22 ± 2 °C and a 16-h photoperiod of light with an intensity of 40 µE m^−2^ s^−1^.

### 4.2. Se Treatment

The experimental framework included for all cultivars the comparison between explants grown with five different concentrations (0, 10, 20, 40, and 80 mg L^−1^) of Na_2_SeO_4_, added as a solution (10 mL for each vessel) filtered on the surface of the medium after the positioning of the explants. For the 0 mg L^−1^ Na_2_SeO_4_ concentration in each vessel, 10 mL of distilled water without Se addition was added. Three subsequent subcultures were carried out, but in the second and the third ones, only the dosages that ensured an adequate number of reusable explants and/or those that determined positive effects on growth in the first subculture were maintained for each variety (Table 6).

For each treatment, a number of vessels (replicates) equal to six was foreseen: this allowed, at the end of the proliferation, to carry out the destructive measurements on the shoots of three pots and to assure the suitable number of explants for the next subcultures.

To evaluate the effect of treatments, at the end of 45 days of each subculture, the following parameters were monitored:

- *viability* (%): incidence of green and viable explants;
- *shoots* (n): average number of shoots developed by each initial explant;
- *shoot length* (mm): average length of developed shoots;
- *nodes* (n): average number of nodes developed by each initial explant, reusable for further proliferation subculture (multiplication rate);
- *callus* (%): incidence of explants that produced basal callus;
- *green fresh weight* (mg): average fresh weight of developed vegetative organs (leaves, stems, buds);
- *callus fresh weight* (mg): average fresh weight of callus masses that may have developed at the base of explants;
- *total dry weight* (mg): average dry weight of the vegetative organs and callus, obtained by keeping the plant material in an oven for three days at 105 °C;
- *dry matter* (%): average incidence of dry matter on total fresh weight, calculated for each comparison (calculated values).

### 4.3. Total Se Content of the Proliferated Explants

At the end of each subculture, the total Se content of the oven dried shoots was determined in the acidic solution (mixture of HNO_3_ and H_2_O_2_ (9:1, *v/v*)) according to the US-EPA method 3052 [45] on three samples (2.0 g each one) for each Se concentration. The determination of the Se in digested materials was accomplished by using an atomic absorption spectrophotometer, equipped with a graphite furnish and a deuterium lamp (Shimadzu AA-6800, GF-AAS, “Shimadzu Corp.”, Tokyo, Japan). The background correction was done using a matrix modifier (Pd(NO_3_)_2_, 0.5 mol M in HNO_3_).

### 4.4. Statistical Analysis

Collected data were subjected for each cultivar to two-way analysis of variance (ANOVA) considering for each cultivar Se concentration and subculture as factors, and significant differences were assayed by Duncan’s test (*p* = 0.05). The details of statistical analysis of the data reported in the figures were included in the Supplementary Materials.

## 5. Conclusions

It is well known that in vitro plant cultures represent an effective tool to support different types of studies, from genetic to physiological and biochemical ones. Indeed, aseptic cultures can assure reliable results in a short time compared to the growth in the field. The results suggest a positive effect of an appropriate amount of Se, although with different intensities depending on the cultivar, on the vigor of the olive tree proliferated shoots in vitro. Obtaining more robust explants is very important for the subsequent rooting and transplantation in ex vitro conditions.

In addition, further studies will be useful to evaluate the possible residual effect of Se absorbed by the shoots during proliferation (as demonstrated by the analysis of total Se content) on the rooting capacity of olive explants.

## Figures and Tables

**Table 1:** Average values of the three subcultures for proliferated explants of the ‘San Felice’ cv.

Na_2_SeO_4_	Vitality	Shoots	Shoots Length	Nodes	Green Fresh Weight	Callus Fresh Weight	Total Dry Weight	Dry Matter
(mg L^−1^)	(%)	(n)	(mm)	(n)	(mg)	(mg)	(mg)	(%)
0 (control)	100 a	2.72 a	62.68 a	11.49 a	316.50 ± 55.4 b	597.94 a	537.83 ± 99.8 b	58.81 ± 7.3 b
20	100 a	2.93 a	80.83 a	12.91 a	351.22 ± 43.8 ab	872.22 a	823.54 ± 130.4 a	67.31 ± 2.7 a
40	100 a	2.80 a	84.59 a	11.90 a	466.22 ± 90.1 a	925.89 a	830.66 ± 187.6 a	59.67 ± 3.2 b

In each column, mean values ± SE followed by different letters were significantly different (*p* < 0.05).

**Table 2:** Average values of the three subcultures for proliferated explants of the ‘Moraiolo’ cv.

Na_2_SeO_4_	Vitality	Shoots	Shoots Length	Nodes	Green Fresh Weight	Callus Fresh Weight	Total Dry Weight	Dry Matter
(mg L^−1^)	(%)	(n)	(mm)	(n)	(mg)	(mg)	(mg)	(%)
0 (control)	100 a	3.29 a	91.84 a	11.71 a	415.89 a	567.11 a	680.69 a	69.25 a
10	100 a	3.80 a	125.22 a	13.69 a	524.78 a	643.33 a	737.95 a	63.17 a
20	100 a	3.67 a	123.22 a	14.13 a	407.00 a	533.57 a	652.16 a	69.33 a

In each column, mean values ± SE followed by different letters were significantly different (*p* < 0.05).

**Table 3:** Average values of the three subcultures for proliferated explants of the ‘Frantoio’ cv.

Na_2_SeO_4_	Vitality	Shoots	Shoots Length	Nodes	Green Fresh Weight	Callus Fresh Weight	Total Dry Weight	Dry Matter
(mg L^−1^)	(%)	(n)	(mm)	(n)	(mg)	(mg)	(mg)	(%)
0 (control)	100 a	1.78 a	49.11 a	6.42 a	156.78 a	698.69 a	572.23 a	66.89 a
10	100 a	1.82 a	58.22 a	7.18 a	150.22 a	616.33 a	545.47 a	71.16 a
20	100 a	1.59 a	50.72 a	5.87 a	117.44 a	476.56 a	413.73 a	69.65 a

In each column, mean values ± SE followed by different letters were significantly different (*p* < 0.05).

**Table 4:** Average values of the three subcultures for proliferated explants of the ‘Canino’ cv.

Na_2_SeO_4_	Vitality	Shoots	Shoot Length	Nodes	Green Fresh Weight	Callus Fresh Weight	Total Dry Weight	Dry Matter
(mg L^−1^)	(%)	(n)	(mm)	(n)	(mg)	(mg)	(mg)	(%)
0 (control)	100 a	3.42 a	76.78 a	13.71 a	355.00 ± 56.5 b	439.56 a	612.58 ± 64.4 a	77.09 a
20	100 a	2.69 a	79.21 a	11.71 a	316.56 ± 39.1 b	395.44 a	466.97 ± 82.5 b	65.59 a
40	100 a	3.92 a	84.59 a	12.80 a	496.89 ± 157.4 a	495.11 a	649.16 ± 106.1 a	65.44 a

In each column, mean values ± SE followed by different letters were significantly different (*p* < 0.05).

**Table 5:** Total Se content in explants (µg kg^−1^ plant DW) at the end of the trial.

	µg Se kg^−1^ Plant DW		
	Subculture 1	Subculture 2	Subculture 3
Na_2_Se O_4_ (mg L^−1^)	‘San Felice’		
0 (control)	0.92 ± 0.1 d	2.65 ± 0.8 c	1.12 ± 0.2 c
10	20.97 ± 3.1 c	-	-
20	31.40 ± 3.4 c	24.79 ± 3.8 b	30.48 ± 3.6 b
40	57.87 ± 4.6 b	53.84 ± 3.4 a	54.27 ± 1.5 a
80	95.54 ± 3.3 a	-	-
	‘Moraiolo’		
0 (control)	1.04 ± 0.2 d	1.60 ± 0.4 b	1.22 ± 0.3 b
10	10.59 ± 2.1 c	11.46 ± 2.6 a	9.83 ± 1.9 a
20	17.00 ± 2.3 c	19.09 ± 1.4 a	19.45 ± 5.5 a
40	33.53 ± 6.8 b	-	-
80	94.85 ± 10.4 a	-	-
	‘Frantoio’		
0 (control)	0.84 ± 0.3 e	1.23 ± 0.4 c	1.20 ± 0.3 c
10	8.55 ± 1.4 d	14.12 ± 2.5 b	12.47 ± 1.8 b
20	31.18 ± 4.2 c	31.74 ± 3.1 a	28.96 ± 5.8 a
40	53.73 ± 2.1 b	-	-
80	130.73 ± 14.6a	-	-
	‘Canino’		
0 (control)	0.99 ± 0.4 d	1.17 ± 0.2 c	1.15 ± 0.4 c
10	19.84 ± 5.6 c	-	-
20	29.02 ± 5.8 c	32.53 ± 3.1 b	28.74 ± 6.1 b
40	63.87 ± 3.5 b	55.90 ± 4.3 a	58.36 ± 6.2 a
80	114.21 ± 18.6 a	-	-

In each column and for each cultivar mean values ± SE followed by different letters were significantly different (*p* < 0.05).

**Table 6:** Na_2_SeO_4_ concentrations used for each cultivar in the second and third subcultures.

Cultivar	Na_2_SeO_4_ (mg L^−1^)				
	0 (Control)	10	20	40	80
‘San Felice’	✓	-	✓	✓	-
‘Moraiolo’	✓	✓	✓	-	-
‘Frantoio’	✓	✓	✓	-	-
‘Canino’	✓	-	✓	✓	-

## Data Availability

The data that support the findings of this study are available from the corresponding author upon reasonable request.

## Supplementary Materials

The following are available online at https://www.mdpi.com/article/10.3390/plants10081630/s1, file supplementary material.

## References

[1] Birringer M., Pilawa S., Flohé L. (2002). Trends in selenium biochemistry. Nat. Prod. Rep..

[2] Germ L.M., Stibilj V., Kreft I. (2007). Metabolic importance of selenium for plants. Eur. J. Plant Sci. Biotechnol..

[3] Zhu Y.G., Pilon-Smits E.A.H., Zhao F.J., Williams P.N., Meharg A.A. (2009). Selenium in higher plants: Understanding mechanisms for biofortification and phytoremediation. Trends Plant Sci..

[4] Hamilton S.J. (2004). Review of selenium toxicity in the aquatic food chain. Sci. Total Environ..

[5] Kapoor R., Nasim S.A., Dhir B., Mujib A. (2012). Selenium treatment alters phytochemical and biochemical activity of in vitro-grown tissues and organs of *Allium sativum* L. In Vitro Cell. Dev. Biol. Plant.

[6] Hartikainen H., Xue T., Piironen V. (2000). Selenium as an antioxidant and prooxidant in ryegrass. Plant Soil.

[7] El-Ramady H., Abdalla N., Taha H.S., Alshaal T., El Henawy A., Salah E.D.F., Shams M.S., Youssef S.M., Shalaby T., Bayoumi Y. (2016). Selenium and nano-selenium in plant nutrition. Environ. Chem. Lett..

[8] Sors T.G., Ellis D.R., Gun N.N., Lahner B., Lee S., Leustek T., Pickering I.J., Salt D.E. (2005). Analysis of sulfur and selenium assimilation in Astragalus plants with varying capacities to accumulate selenium. Plant J..

[9] Iwaoka M., Ooka R., Nakazato T., Yoshida S., Oishi S. (2008). Synthesis of selenocysteine and selenomethionine derivatives from sulfur-containing aminoacids. Chem. Biodivers..

[10] Zhou X., Yang J., Kronzucker H.J., Shi W. (2020). Selenium Biofortification and Interaction with Other Elements in Plants: A Review. Front. Plant Sci..

[11] Kuznetsov V., Kholodova V., Kuznetsov V., Yagodin B.A. (2003). Selenium regulates the water status of plants exposed to drought. Dokl. Biol. Sci..

[12] Malerba M., Cerana R. (2018). Effect of Selenium on the Responses Induced by Heat Stress in Plant Cell Cultures. Plants.

[13] Pezzarossa B., Remorini D., Gentile M.L., Massai R. (2012). Effects of foliar and fruit addition of sodium selenate on selenium accumulation and fruit quality. J. Sci. Food Agric..

[14] Proietti P., Nasini L., Del Buono D., D’Amato R., Tedeschini E., Businelli D. (2013). Se protects olive (*Olea europaea* L.) from drought stress. Sci. Hortic..

[15] D’Amato R., De Feudis M., Hasuoka P.E., Regni L., Pacheco P.H., Onofri A., Businelli D., Proietti P. (2018). The selenium supplementation influences olive tree production and oil stability against oxidation and can alleviate the water deficiency effects. Front. Plant Sci..

[16] Regni L., Palmerini C.A., Del Pino A.M., Businelli D., D’Amato R., Mairech H., Marmottini F., Micheli M., Pacheco P.H., Proietti P. (2021). Effects of selenium supplementation on olive under salt stress conditions. Sci. Hortic..

[17] D’Amato R., Proietti P., Nasini L., Del Buono D., Tedeschini E., Businelli D. (2014). Increase in the selenium content of extra virgin olive oil: Quantitative and qualitative implications. Grasas Aceites.

[18] D’Amato R., Proietti P., Onofri A., Regni L., Esposto S., Servili M., Businelli D., Selvaggini R. (2017). Biofortification (Se): Does it increase the content of phenolic compounds in virgin olive oil (VOO)?. PLoS ONE.

[19] Tedeschini E., Proietti P., Timorato V., D’Amato R., Nasini L., Del Buono D., Frenguelli G. (2015). Selenium as stressor and antioxidant affects pollen performance in *Olea europaea*. Flora Morphol. Distrib. Funct. Ecol. Plants.

[20] Del Pino A.M., Regni L., D’Amato R., Tedeschini E., Businelli D., Proietti P., Palmerini C.A. (2019). Selenium-enriched pollen grains of *Olea europaea* L.: Ca^2+^ signalling and germination under oxidative stress. Front. Plant Sci..

[21] D’Amato R., Petrelli M., Proietti P., Onofri A., Regni L., Perugini D., Businelli D. (2018). Determination of changes in the concentration and distribution of elements within olive drupes (cv. Leccino) from Se biofortified plants, using laser ablation inductively coupled plasma mass spectrometry. J. Sci. Food Agric..

[22] D’Amato R., Regni L., Falcinelli B., Mattioli S., Benincasa P., Dal Bosco A., Pacheco P., Proietti P., Troni E., Santi C. (2020). Current Knowledge on Selenium Biofortification to Improve the Nutraceutical Profile of Food: A Comprehensive Review. J. Agric. Food Chem..

[23] Kavand S., Kermani M.J., Haghnazari A., Khosravi P., Azimi M.R. (2011). Micropropagation and medium-term conservation of Rosa pulverulenta. Acta Sci. Agron..

[24] Balla I., Mansvelt L. (2013). Micropropagation of peach rootstocks and cultivars. Methods Mol. Biol..

[25] Dobránszki J., Silva J.A. (2010). Micropropagation of apple—A review. Biotechnol. Adv..

[26] Mangal M., Sharma D., Sharma M., Kumar S. (2014). In vitro regeneration in olive (*Olea*
*europaea* L.), cv “Frantoio” from nodal segments. Indian J. Expt. Biol..

[27] Micheli M., El Behi A.W., Zakhour D., Yasin M., Standardi A. (2010). In vitro proliferation of olive (‘Dolce Agogia’ and “Moraiolo”): Effect of different cytokinins. Acta Hort..

[28] Breznik B., Germ M., Gaberščik A., Kreft I. (2004). Combined effects of elevated UV-B radiation and the addition of selenium on common (*Fagopyrum esculentum* Moench) and tartary [*Fagopyrum tataricum* (L.) Gaertn.] buckwheat. Photosynthetica.

[29] Van Hoewyk D. (2013). A tale of two toxicities: Malformed selenoproteins and oxidative stress both contribute to selenium stress in plants. Ann. Bot..

[30] Dimkovikj A., Fisher B., Hutchison K., Van Hoewyk D. (2015). Stuck between a ROS and a hard place: Analysis of the ubiquitin proteasome pathway in selenocysteine treated Brassica napus reveals different toxicities during selenium assimilation. J. Plant Physiol..

[31] Van Hoewyk D. (2016). Defects in endoplasmic reticulum-associated degradation (ERAD) increase selenate sensitivity in Arabidopsis. Plant. Signal. Behav..

[32] Kolbert Z., Lehotai N., Molnár A., Feigl G. (2016). “The roots” of selenium toxicity: A new concept. Plant. Signal. Behav..

[33] Fritz W.P., Spracklen R.E., Spiby M.C., Meacham A., Mead M., Harriman L.J., Trueman B.M., Smith B., Thomas M.R. (2004). Interactions between selenium and sulphur nutrition in Arabidopsis thaliana. J. Exp. Bot..

[34] Wrobel K., Esperanza M.G., Barrientos E.Y., Escobosa A.R.C., Wrobel K. (2020). Different approaches in metabolomic analysis of plants exposed to selenium: A comprehensive review. Acta Physiol. Plant..

[35] Gupta M., Gupta S. (2017). An Overview of Selenium Uptake, Metabolism, and Toxicity in Plants. Front. Plant Sci..

[36] Winkel L.H.E., Vriens B., Jones G.D., Schneider L.S., Pilon-Smits E.A.H., Banuelos G.S. (2015). Selenium cycling across soil–plant–atmosphere interfaces: A critical review. Nutrients.

[37] Germ M., Kreft I., Stibilj V., Urbanc-Berčič O. (2007). Combined effects of selenium and drought on photosynthesis and mitochondrial respiration in potato. Plant. Physiol. Biochem..

[38] Garcia-Ferrìz L., Ghorbel R., Ybarra M., Mari A., Belay A., Trujillo I. (2002). Micropropagation of olive mature trees. Acta Hort..

[39] Sgir S., Chatelet P., Ouazzani N., Dosba F., Belkoura I. (2005). Micropropagation of eight moroccan and french olive cultivars. Hortscience.

[40] Ali A., Ahmad T., Abbasi N.A., Hafiz I.A. (2009). Effect of different media and growth regulators on in vitro shoot proliferation of olive cultivar “Moraiolo”. Pak. J. Bot..

[41] Mendoza-de Gyves E., Mira F.R., Ruiu F., Rugini E. (2008). Stimulation of node and lateral shoot formation in micropropagation of olive (*Olea europaea* L.) by using dikegulac. Plant Cell Tissue Organ. Cult..

[42] Peixe A., Raposo A., Lourenco R., Cardoso H., Macedo A. (2007). Coconut water and BAP successfully replaced zeatin in olive (*Olea europaea* L.) micropropagation. Sci. Hort..

[43] Demo P., Kuria P., Nyende A.B., Kahangi E.M. (2008). Table sugar as an alternative low cost medium component for in vitro micropropagation of potato. Afr. J. Biotechnol..

[44] Rugini E. (1984). In vitro propagation of some olive (*Olea europea* L.) cultivars with different root-ability, and medium development using analytic data from developing shoots and embryos. Sci. Hort..

[45] US EPA Method 3052 Microwave Assisted Acid Digestion of Siliceous and Organically Based Matrices. https://www.epa.gov/hw-sw846/sw-846-test-method-3052-microwave-assisted-acid-digestion-siliceous-and-organically-based.

